# Prolongation anormale d'un bloc fémoral analgésique: cas Clinique

**DOI:** 10.11604/pamj.2015.22.190.7300

**Published:** 2015-10-26

**Authors:** Joseph Koné, Mustapha Bensghir, El Houcine Boutayeb, Charki Haimeur

**Affiliations:** 1 Pôle Anesthésie Réanimation Hôpital Militaire Med V Rabat 1, Faculté de Médecine et de Pharmacie de Rabat, Université Med V Souissi, Rabat, Maroc

**Keywords:** Bloc nerveux fémoral, dexamethasone, prolongation anormale, femoral nerve block, dexamethasone, abnormal extension

## Abstract

La prolongation anormale d'un bloc nerveux peut être définie comme un dépassement du délai habituel de récupération sensitive ou motrice. A travers un cas clinique d'une prolongation anormale d'un bloc analgésique et une revue de la littérature, les auteurs discutent les facteurs de risque et les moyens de prévention de cette complication.

## Introduction

L'anesthésie locorégionale périphérique connait de plus en plus de développements aussi bien au niveau des techniques de repérage, du matériel que dans l'utilisation des agents anesthésiques [1]. De nombreux adjuvants aux anesthésiques locaux sont utilisés, comme l'adrénaline, le sulfate de magnésium, la clonidine, la morphine ou la dexaméthasone [2, 3]. L'utilisation de ces agents adjuvants en combinaison avec les anesthésiques locaux a largement démontré son efficacité avec toute fois des complications propres [4]. Certaines complications sont décrites suite aux blocs nerveux périphériques comme les ponctions vasculaires accidentelles, les atteintes nerveuses transitoires [5–7]. Nous rapportons une prolongation inhabituelle de la durée d'un bloc analgésique chez une patiente suivie pour une cruralgie chronique. A travers ce cas, et une revue de la littérature nous discutons les causes de prolongation anormale, les mesures préventives et la conduite à tenir devant les situations à risque.

## Patient et observation

Il s'agissait d'une patiente de 42 ans sans antécédents particuliers consultant en urgence pour une crise hyperalgique d'une cruralgie chronique unilatéral du membre inférieur droit. Elle présentait une allodynie touchant la fesse, la face antérieure de la cuisse, la face antérieure de la jambe sans déficit moteur et n'ayant pas cédé aux antalgiques et d'anti-inflammatoires non stéroïdiens. Le reste de l'examen physique était normal tant au niveau lombaire qu'au membre inférieur gauche. Après asepsie soigneuse, un bloc analgésique du nerf crural a été réalisé. Le repérage du nerf a été fait par la technique de neurostimulation de Winnie avec une intensité minimale de stimulation de 0,35 mA, suivi d'une injection fractionnée d'un mélange de 4 ml de bupivacaïne à 0,125% et de 1 ml (soit 4mg) de dexaméthasone soit un volume total de 5ml. Aucun incident de type paresthésie n'a été noté durant la réalisation du bloc. L'installation d'un bloc sensitif dans le territoire d'innervation du nerf crural s’était faite dans un délai de vingt minutes ainsi qu'un bloc moteur profond d'où la mise en place d'un protocole de suivi horaire. Nous avons observé une persistance du bloc moteur pendant dix-huit heures, une récupération sensitive au bout de vingt-trois heures. Devant cette prolongation anormale du bloc analgésique, une échographie des parties molles était faite. Aucune anomalie locale n’était notée.

## Discussion

La constatation d'une prolongation anormale d'un bloc nerveux anesthésique est une situation inquiétante qui nécessite une démarche diagnostique et thérapeutique rigoureuse. Peut être défini comme anormal, un dépassement du délai habituel de récupération sensitive ou motrice en fonction de la durée d'action de l'anesthésique local utilisé et du contexte clinique (propriétés physico-chimiques, volume, concentration, etc). La prolongation de la durée du bloc est souvent un but recherché justifiant l'utilisation des adjuvants avec les anesthésiques locaux. Une longue durée d'analgésie permet une meilleure gestion des suites post-opératoires comme la mobilisation précoce, les man'uvres de rééducation, et la réduction des nausées vomissements par épargne en morphiniques [8]. Cependant l'absence de récupération motrice et sensitive dans les délais habituels suscite des craintes de complications. [9] C'est le cas de notre patiente dont le bloc a perduré au-delà de 23 heures, et qui surtout a présenté un bloc moteur, nous poussant à rechercher les causes possibles. Un bloc prolongé de façon anormale peut être dû à plusieurs causes possibles dont une erreur dans la dose de l'anesthésique local injecté, une pathologie neurale préexistante d'origine ischémique, métabolique, inflammatoire ou même iatrogène [10–13]. Notre patiente avait une sciatique avec une allodynie sous-entendant une souffrance chronique. Le nerf pathologique a fait l'objet d’études dans la pratique des blocs nerveux périphériques. Les pathologies de la gaine de myéline, les pathologies dégénératives du système nerveux central ou de la moelle épinière peuvent induire une prolongation anormale d'un bloc périphérique ou tronculaire [14]. Les caractéristiques propres au patient ont été associées à une prolongation de la durée de bloc. Dans une étude multicentrique sur 5143 patients, une analyse multi variée a mis en évidence que l’âge supérieur à 65ans, (OR:1,2), l'artériopathie (OR:2), l'existence d′une pathologie inflammatoire (OR:1,9), une neuropathie périphériques avérée (OR:1,9) et une neuropathie médullaire (OR:2,1) sont des facteurs de prolongation qui peut être considérée comme anormale de la durée d′un bloc périphérique [15]. De même, le délai de récupération nerveuse parait plus long chez les patients ayant une neuropathie diabétique (p < 0,01) [16]. L’âge du patient est aussi rapporté comme ayant une forte corrélation statistique avec la longue durée des blocs (r= 0,56; p< 0.05); avec une différence significative entre les sujets de 77 ans comparés à ceux de 39 ans [17]. Le rôle des adjuvants associés aux anesthésiques locaux est démontré dans la qualité des blocs, toutefois leur innocuité reste à évaluer [18]. La dexaméthasone utilisée comme adjuvant chez notre patiente, a fait l'objet de nombreuses études avec une prolongation significative de la durée du bloc anesthésique [8]. Dans notre cas, il s'agissait d'un nerf pathologique prédisposé à des troubles de la conduction de l'influx nerveux du fait de la souffrance chronique. La concentration de l'anesthésique local était très faible excluant l'hypothèse de toxicité propre. L’échographie de contrôle n'avait retrouvé ni signe de compression locale, ni d'hématome, ni œdème intra tronculaire. Le nerf controlatéral était d’écho structure similaire. La puissance anesthésique de la dexaméthasone et le profil pathologique du nerf pourraient être retenus comme étant les facteurs de prolongation anormale que nous avons observée chez notre patiente. L'atteinte nerveuse préexistante parait être un facteur capital pour une prolongation anormale. L’échographie en permettant une maîtrise des volumes administrés, reste la meilleure technique de pratique des blocs nerveux [19, 20]. Il convient sur le nerf pathologique de diminuer les doses (volume et concentration) de l'anesthésique local qu'il soit utilisé pur ou avec adjuvant, et d'assurer un monitorage du bloc.

## Conclusion

La prolongation anormale d'un bloc nerveux au-delà des délais habituels est une situation alarmante. La pratique de l'anesthésie locorégionale périphérique nécessite un examen pré-anesthésique dont la recherche d'une souffrance nerveuse préexistante, une technique de repérage sécurisée, et un monitorage des fonctions nerveuses.
